# Firearm safe storage practices among firearm owners in rural and urban households

**DOI:** 10.1186/s40621-025-00587-9

**Published:** 2025-06-13

**Authors:** Yi-Fang Lu, Victor A. Soupene

**Affiliations:** 1 Center for Social Science Innovation, Injury Prevention Research Center, University of Iowa, 605 E Jefferson Street, Iowa City, IA 52242 USA; 2 Department of Occupational and Environmental Health, Department of Emergency Medicine, University of Iowa, 145 N Riverside Dr S331, Iowa City, IA 52242 USA

**Keywords:** Firearm, Storage, Rural, Non-metropolitan, BRFSS

## Abstract

**Background:**

Safe firearm storage may reduce suicide and unintentional firearm injuries. However, little is known about safe storage practices in rural US populations. We aimed to examine the association between living in a rural US area and firearm storage practices.

**Methods:**

We used data from the Behavioral Risk Factor Surveillance System (BRFSS), 2021–2023 to compare firearm storage practices between rural and urban populations. The primary outcomes were whether firearms were stored loaded, and among loaded firearms, whether they were stored unlocked. Those living in a rural residence were compared to those living in an urban residence. Descriptive statistics for firearm storage practices were compared between living in rural or urban areas. Unadjusted and adjusted relative risks (with 95% confidence intervals) were calculated using Poisson regression models with robust standard errors.

**Results:**

One third of rural (34.71%) and urban (34.33%) firearm-owning households stored at least one firearm loaded. Of these households with loaded firearm(s), 58.55% of rural respondents stored their firearms unlocked compared to 50.66% of urban respondents. Compared to the urban sample, rural respondents were older adults (51.41% vs. 43.91% ≥ the age of 55), non-Hispanic White (83.12% vs. 72.45%), and were high school graduates or less (48.33% vs. 34.77%). While rural respondents were equally likely as urban respondents to store firearms loaded (ARR = 1.00, CI = [0.93-1.06]), they were more likely to store loaded firearms unlocked (ARR = 1.11, CI = [1.03–1.19]).

**Conclusions:**

Additional support for providing firearm storage options, such as firearm safety locks, may promote safer firearm storage practices in rural populations. Future research should explore culturally appropriate interventions tailored to the specific needs of individuals living in the rural US.

**Supplementary Information:**

The online version contains supplementary material available at 10.1186/s40621-025-00587-9.

## Background

Firearms contributed to the second highest age-adjusted rate for fatal injuries–behind drug poisonings–in the US in 2022 with firearm suicides and homicides making up 96.9% of these fatal injuries [1]. Firearm injuries appear different between rural and urban counties. While the firearm injury rate is higher among rural (also known as non-metropolitan) counties (18.0 per 100,000) compared urban (also known as metropolitan) counties (13.7 per 100,000), the rate in rural US counties is led by firearm suicides (12.0 per 100,000) compared to urban counties (6.9 per 100,000) [1]. The disparities in firearm injury rates between urban and rural areas have remained consistent over the past two decades [2]. Further, rates of unintentional firearm injuries were found to be higher in rural counties than in urban counties [3, 4]. Previous literature has suggested firearm-related injuries are particularly high in rural areas likely due to greater access to firearms for hunting or social connections [5, 6, 7, 8, 9] and less safe storage of firearms [10, 11].

Few studies have examined safe storage practices of firearms in rural areas with mixed findings [9, 10, 11, 12, 13, 14]. Two studies focusing on firearm storage among US households found that suburban and rural firearm owners with children were as likely to report unsafe firearm storage as urban firearm owners [14, 15]. Studies examining adolescents in Future Farmers of America found that adolescents who lived on farms were more likely to have used rifles/shotguns and handguns compared to those who lived in towns [9], and among those who had access to firearms, those who lived on farms were more likely to have at least one firearm in the home be stored unlocked and/or loaded compared to those who lived in towns [11]. However, these findings are based on adolescents’ knowledge of where and how firearms were stored in their homes and are limited to households situated on farms or in towns. Indeed, more research is needed to understand firearm storage practices among US adults in rural areas compared to urban areas, particularly after a drastic surge in firearm purchases during the COVID-19 pandemic in the US [16].

Safe and secure firearm storage is the primary method of reducing access to firearms. Best practices for firearm storage involve storing firearms locked, unloaded, and separate from ammunition—preferably locked up [13, 17, 18]. Many states have implemented child access prevention (CAP) laws to regulate firearm storage in households with minors. State- or macro-level analyses indicate that stringent CAP laws are associated with reductions in pediatric firearm homicides, suicides, and fatal and nonfatal unintentional injuries [19, 20, 21, 22]. Additionally, it is well documented that those who stored firearms locked, unloaded, or both were less likely to die from firearm suicide or unintentional firearm injuries compared to those who stored their firearm unlocked and loaded [23, 24, 25]. Although studies have highlighted the critical importance of safe and secure firearm storage, it is estimated that less than half of firearm owners or firearm-owning households with children in the US stored all their firearms locked and unloaded [15, 26, 27]. Two national surveys estimated that approximately 4.6 million US children have access to at least one loaded and unlocked firearm in their home [15, 26]. These is also evidence showing that adolescent handgun carrying rates are higher in rural areas compared to urban areas likely due to higher firearm access [28]. Nevertheless, there is limited knowledge regarding firearm storage practices in the rural US where suicides and unintentional firearm deaths are more prevalent.

The purpose of this study is to examine firearm storage practices among adults in rural and urban areas in the US. Given the higher rates of firearm ownership and firearm suicides in rural areas, it is crucial to assess the extent to which firearms are stored in a safe manner among rural firearm owners to prevent firearm injuries. Identifying modifiable risk factors related to firearms is essential for informing interventions and prevention strategies tailored to the needs of rural firearm-owning households.

## Methods

We used survey data from the Behavioral Risk Factor Surveillance System (BRFSS), 2021–2023. The BRFSS is a nationwide public health surveillance system that collects annual data by telephone surveys about US residents’ health-related risk factors, chronic health, and preventive health practices. Eligible respondents were adults living in randomly selected private households. More than 400,000 noninstitutionalized adults participated in the interviews each year. The BRFSS survey consists of core modules and optional modules. Core modules include standard questions that were asked by all US states, covering a wide variety of health-related issues and risk factors as well as demographic information. Optional modules include standardized questions on various topics that each state may select to include in their questionnaire. From 2021 to 2023, the BRFSS included an optional module on firearm safety. In this module, respondents were asked about household firearm ownership and firearm storage practices. Specifically, respondents were first asked whether there were any firearms kept in or around their home. If there were any firearms in or around their home, they were then asked whether any of these firearms were loaded. If there were loaded firearms, they were asked whether any of these loaded firearms were also unlocked. Respondents may or may not have been the firearm owners themselves. The sample of this study consisted of respondents with firearm(s) in households in the 19 states that selected to administer the firearm safety module in 2021 (Alaska, California, New Mexico, North Carolina, Ohio, and Oklahoma), 2022 (California, Minnesota, Neveda, New Mexico, and Ohio), and 2023 (Arizona, Idaho, Indiana, Louisiana, Michigan, Neveda, New Jersey, New Mexico, Oregon, South Carolina, Vermont, Virginia, and West Virginia).

The outcome of this study is firearm storage. Specifically, two dichotomous variables were used to indicate (1) whether firearm owners kept their firearm loaded and (2) whether the loaded firearms were unlocked. For the independent variable, we used the urban/rural indicator in the BRFSS. The BRFSS defines rural counties as non-metropolitan counties that did not qualify as micropolitan (micropolitan urban cluster population between 10,000 and 49,999) using the 2013 National Center for Health Statistics Urban-Rural Classification Scheme for US counties [29]. In other words, we compared respondents from firearm-owning households in *rural* counties (also known as *noncore* counties) to respondents from firearm-owning households in *metropolitan* and *micropolitan* counties. As a supplementary analysis, we also used a broader rural classification scheme: metropolitan counties compared to non-metropolitan, where counties classified as micropolitan and noncore counties were considered non-metropolitan.

The covariates included in this analysis were sex (male or female), age group (18–24, 25–34, 35–44, 45–54, 55–64, or 65+), race/ethnicity (non-Hispanic White, non-Hispanic Black, non-Hispanic other, or Hispanic), marital status (married, divorce/separated, widowed, never married, or member of an unmarried couple), education attainment (less than high school, high school graduate, some college, or college graduate), employment status (employed or self-employed, out of work, homemakers, students, retired, or unable to work), any children less than 18 years of age in home (yes or no), having ever served on active duty in the US military (yes or no) [30], and any binge drinking in the past 30 days prior to the interview (yes or no) [10, 31, 32]. All covariates were measured in the core module.

To account for the complex sampling design for multiple years of data, we followed CDC’s instructions for preparing a module dataset for analysis to adjust the sampling weights for all the states across three years [33]. Specifically, the original sampling weight in the BRFSS dataset was proportionately adjusted based on state participation by year. For instance, New Mexico included the firearm safety module in 2021 (*n* = 6,362), 2022 (*n* = 4,758), and 2023 (*n* = 3,220), yielding a total sample of 14,340. For New Mexico respondents, the initial weights were multiplied by 0.44 (6,362/14,340) for the 2021 sample, by 0.33 (4,758/14,340) for the 2022 sample, and by 0.22 (3,220/14,340) for the 2023 sample.

We reported descriptive statistics including unweighted frequencies and weighted percentages for all variables by rural and urban designation. Given that the outcomes of interest are common (i.e., > 30% prevalence), we used Poisson regression models with robust standard errors to examine the association between living in a rural area and unsafe firearm storage practices [34, 35]. Two separate sets of models were performed to examine (1) the estimated risk of storing firearms loaded and (2) the estimated risk of storing loaded firearms unlocked between rural and urban respondents. Relative risks (unadjusted [RR] and adjusted [ARR]) with 95% confidence intervals (CIs) were reported with covariates including sex, age, race/ethnicity, marital status, education attainment, employment status, veteran, children in home, and binge drinking as potential confounders. In all Poisson regression models, we included fixed effects of state and interview year to account for geographical/contextual and temporal variations. Since a small proportion of BRFSS interviews (3.7 to 7.5%) were conducted in the first two months of the following year, interview years range from 2021 to 2024 (2021 as the reference year). Given that the percentages of missing are relatively low (see Table 1), we used listwise deletion to handle missing data. We conducted the data analysis using Stata 15 with the *svyset* command to adjust for the sampling design (StataCorp LLC, College Station, TX, USA).

**Table 1:** Distributions of demographic characteristics, risk factors, state, and year among rural and urban study participants (*n* = 60,548)

	Urban		Rural	
	Unweighted frequency	Weighted %	Unweighted frequency	Weighted %
**Firearm households total**	52,002	91.08	8,546	8.92
**Firearm storage**				
Loaded	17,204	34.33	2,833	34.71
Unloaded	33,155	65.67	5,467	65.29
Don’t know/refused/missing	1,643	-	246	-
**Loaded firearm**				
Unlocked	9,293	50.66	1,722	58.55
Locked	7,724	49.34	1,075	41.45
Don’t know/refused/missing	187	-	36	-
**Sex**				
Male	27,870	55.64	4,384	53.43
Female	24,132	44.36	4,162	46.57
**Age group**				
18–24	2,577	10.05	332	9.37
25–34	4,768	14.75	576	11.32
35–44	6,619	15.97	971	14.30
45–54	7,788	15.31	1,097	13.60
55–64	10,372	18.00	1,822	20.12
65+	19,343	25.91	3,674	31.29
Missing	535	-	74	-
**Race/ethnicity**				
Non-Hispanic White	43,207	72.45	7,398	83.12
Non-Hispanic Black	2,668	9.39	225	5.30
Non-Hispanic other race	2,940	8.48	667	7.04
Hispanic	3,187	9.69	256	4.54
**Marital status**				
Married	32,090	60.38	5,330	60.72
Divorced/separated	6,427	10.57	1,062	10.41
Widowed	4,827	5.70	992	8.77
Never married	6,519	18.84	876	16.32
Unmarried couple	1,915	4.51	255	3.77
Missing	224	-	31	-
**Education attainment**				
Less than high school	1,778	6.69	445	12.30
High school graduate	12,953	28.08	2,630	36.03
Some college	15,472	34.91	2,612	32.65
College graduate	21,706	30.32	2,838	19.03
Missing	93	-	21	-
**Employment status**				
Employed or self-employed	27,215	58.77	4,158	52.10
Out of work	1,545	4.17	256	4.42
Homemakers	1,855	4.24	347	5.38
Students	821	3.18	77	2.79
Retired	17,759	24.66	3,151	26.81
Unable to work	2,532	4.98	521	8.50
Missing	275	-	36	-
**Any children less than 18 years of age in household**				
Yes	13,322	33.28	1,997	31.36
No	38,372	66.72	6,513	68.64
Missing	308	-	36	-
**Ever served on active duty in the US military**				
Yes	8,647	15.19	1,306	12.95
No	43,296	84.81	7,231	87.05
Missing	59	-	9	-
**Any binge drinking in past 30 days**				
Yes	8,093	17.97	1,182	15.67
No or no drinking in past 30 days/missing	43,909	82.03	7,364	84.33
**State**				
Alaska	1,454	0.71	1,097	2.85
Arizona	3,520	6.46	353	1.18
California	1,740	16.34	48	4.70
Idaho	2,296	2.39	802	3.50
Indiana	3,174	6.53	329	7.99
Louisiana	1,873	5.11	208	5.06
Michigan	2,667	8.74	262	11.09
Minnesota	4,105	4.58	875	9.08
Nevada	1,669	2.68	74	0.60
New Jersey	883	2.96	0	0
New Mexico	3,971	1.80	352	1.23
North Carolina	1,403	9.90	140	9.10
Ohio	11,097	9.99	1,196	6.40
Oklahoma	2,314	3.48	606	7.28
Oregon	1,713	4.00	83	1.92
South Carolina	3,339	5.73	353	4.47
Vermont	1,700	0.53	784	2.25
Virginia	1,481	5.89	445	13.67
West Virginia	1,603	2.19	539	7.61
**Interview year**				
2021	12,735	23.43	2,522	22.89
2022	12,934	21.74	1,721	17.20
2023	25,399	53.11	4,182	57.76
2024	934	1.72	121	2.14

## Results

Of the 199,494 firearm safety module respondents, 60,548 reported having firearms in or around their households, while 90,248 respondents reported not having firearms in or around their households. The rest of the respondents were categorized as either “don’t know,” or “refused,” or “missing” for the firearm ownership screening question (24.43% of urban and 24.30% of rural respondents). The final sample consists of 8.92% rural respondents from firearm-owning households (unweighted *n* = 8,546) and 91.08% urban respondents from firearm-owning households (unweighted *n* = 52,002) (Table 1). About one in three respondents from firearm-owning households in rural counties (34.71%) and urban counties (34.33%) reported storing at least one loaded firearm. Of those who kept loaded firearm(s), 58.55% of rural respondents stored their loaded firearm unlocked, while 50.66% urban respondents stored their loaded firearm unlocked. Compared to the urban sample, rural respondents on average were older (51.41% vs. 43.91% ≥ the age of 55), non-Hispanic White (83.12% vs. 72.45%), retired (26.81% vs. 24.66%), unable to work (8.05% vs. 4.98%), or had lower levels of education attainment (12.30% vs. 6.69% had less than high school degree, and 36.03% vs. 28.08% were high school graduates).

Table 2 presents the results of Poisson regression models with robust standard errors. The unadjusted model shows that rural respondents were not more likely to keep their firearms loaded than urban respondents (RR = 1.01, *p* =.75, CI = [0.95–1.08]) (Table 2). The effect of rurality on the estimated risk of storing firearms loaded remains non-significant after adjusting for sex, age, race/ethnicity, marital status, education attainment, employment status, veteran, children in home, and binge drinking (ARR = 1.00, *p* =.90, CI = [0.93–1.06]). The results of the adjusted model for the covariates are included in Supplemetary Appendix A. Demographic characteristics with a higher estimated risk for storing firearms loaded include older populations aged 25–34, 35–44, 35–54, 55–64 (vs. 18–24), males, being divorced/separated or widowed (vs. married), non-Hispanic Black (vs. non-Hispanic White), having served in the US military, employed/self-employed (vs. out of work, homemakers, or students), unable to work (vs. employed/self-employed), households without child, and binge drinking in the past 30 days.

**Table 2:** Unadjusted relative risk (RR) and adjusted relative risk (ARR) with 95% confidence intervals examining the association between firearm storage practices and rurality

	Loaded vs. unloaded				Unlocked vs. locked			
	RR	95% CI	ARR	95% CI	RR	95% CI	ARR	95% CI
Urban (reference)	-	-	-	-	-	-	-	-
Rural	1.01	0.95–1.08	1.00	0.93–1.06	1.16***	1.08–1.24	1.11**	1.03–1.19
Unweighted N	58,659		57,255		19,814		19,288	

Note. **p <.01, ***p <.001. RR = relative risk, ARR = adjusted relative risk (adjusted for sex, age, race/ethnicity, marital status, education attainment, employment status, military service, children in home, binge drinking, state, and interview year). The sample is limited to those who stored their firearms loaded in the unlocked vs. locked models

Among those who stored firearms loaded, rural respondents were more likely to store them unlocked than urban respondents (RR = 1.16, *p* <.001, CI = [1.08–1.24]) (Table 2). The effect of rurality on the estimated risk of storing loaded firearms unlocked remains significant after adjusting for covariates—rural respondents were 11% more likely to store their loaded firearms unlocked than their urban counterpart (ARR = 1.11, *p* =.004, CI = [1.03–1.19]). The results of the adjusted model for the covariates are included in Supplementary Appendix B. In addition to rurality, other demographic characteristics with higher estimated risk include age groups over 45 (vs. 18–24), males, being divorced/separated, widowed, or never married (vs. married), retired (vs. employed/self-employed), households without child, and binge drinking in the past 30 days.

From our supplementary analyses, non-metropolitan respondents (including respondents from firearm-owning households in micropolitan and noncore counties) were not more likely to keep their firearms loaded than metropolitan respondents (ARR = 0.98, *p* =.28, CI = [0.93–1.02]) (Supplementary Appendix C). However, they were 11% more likely to keep their loaded firearms unlocked than their metropolitan counterpart (ARR = 1.11, *p* <.001, CI = [1.06–1.17]).

## Discussion

This study examined geographic differences in firearm storage practices using BRFSS data from 2021 to 2023. While rural firearm-owning households were not more likely to keep their firearms loaded compared to urban firearm-owning households, of those who stored firearms loaded, rural households were more likely to keep them unlocked compared to their urban counterpart. Similar findings were revealed when we expanded the rural sample to include micropolitan firearm-owning households. We also found that populations aged 45–64 years, males, those who were divorced/separated or widowed, households without children, and those who reported binge drinking in the past 30 days were at elevated risk of unsafe firearm storage. Those who were retired were more likely to store loaded firearms unlocked compared to employed/self-employed respondents.

A survey of US households with children showed that the odds of keeping at least one firearm loaded and unlocked (vs. no firearm loaded and unlocked) is not significantly higher for suburban/rural firearm owners (as opposed to urban firearm owners) after controlling for the covariates [15]. While their study compared unsafe storage practice (loaded and unlocked) to the safest storage practice (unloaded and locked) and omitted other categorizations of mixed security firearm storage, the difference in our study was drawn from the comparison of unlocked vs. locked loaded firearms (i.e., unsafe vs. one of the mixed security storage practices) in urban and rural areas. The selection of households with children in the previous study might also have yielded a more homogeneous pattern toward safe firearm storage within the sample. Our findings on the higher risk of keeping loaded firearms unlocked in rural areas may be explained by the types of firearms kept in households. Handguns are more common among urban firearm owners, whereas long guns are more prevalent among rural firearm owners [36]. The prevalence of long guns in rural areas may pose greater challenges for safe storage including access to suitable and affordable storage devices to lock up long guns. Moreover, rural firearm owners might have more familiarity and knowledge of firearms and therefore might perceive the need for locking firearms and the risk of firearm access differently from urban firearm owners.

This study highlights the elevated risk of unsecure storage among rural firearm owners. The higher prevalence of unlocked and loaded firearms in rural households might increase the likelihood of access to loaded firearms in households, which might in turn contribute to higher firearm injuries or firearm-carrying among rural youth. How firearms are stored might also affect adolescents’ attitudes toward safe firearm storage. As noted in a study examining rural youth, unsafe storage of long guns and handguns in the home is associated with a lower level of support for safe storage regulations among adolescents [12]. The underestimation of risk might reduce motivation for safe storage, thereby resulting in unsafe storage practices. A national survey shows that nearly half of firearm owners who did not lock all their firearms believed that locking devices were not necessary [37]. Additionally, we identified several demographic characteristics with higher risk of unsafe storage including males, being divorced/separated or widowed, households without children, and alcohol misuse, which are aligned with prior research [13, 30, 32]. The results regarding the elevated risk among middle-aged populations (45–64) are less clear in previous findings. This age group might perceive a greater need for quick access or might not be fully aware of firearm safety recommendations, which merits further investigation.

Messaging and interventions aimed at promoting the safe storage of firearms should be considerate of sub-populations within the rural US, particularly older, non-Hispanic White adults, who comprised of most of the rural study sample and are at higher risk for firearm suicide [1, 38]. The rural sample also largely consisted of those with lower levels of educational attainment, suggesting that messages should be tailored to simpler language to ensure comprehension. Future research should examine how messaging and interventions differ across rural sub-populations and how these efforts can be adapted to incorporate cultural norms and attitudes in rural communities. While suicide prevention strategies are often implemented through clinicians and educators and are effective in some populations [39], a previous study suggests clinicians and educators are not trusted individuals for addressing firearm safety in rural areas compared to other individuals such as law enforcement [40]. One way of improving messaging is by including rural populations in the decision-making process of the intervention. For example, *Store Safely* is a firearm safety program which attempts to consider the cultural norms and values of rural communities through community partnership [41]. Future investigations should continue to examine how cultural norms influence firearm safe storage practices and consider community-participatory research to improve the effectiveness of interventions in the rural US.

Although this study used a large sample of firearm-owning households from 19 states from recent BRFSS data, the findings of this research should be considered with some limitations. First, not all US states were included in the firearm safety module from 2021 to 2023; thus, results can only be generalized to the included US states. Second, we were unable to analyze the locked status of unloaded firearms due to the wording of questions in the BRFSS. This limitation restricts our ability to compare our findings directly to previous studies, particularly regarding the prevalence rates of different firearm storage practices. However, we are still able to understand if individuals store firearms unsafely (i.e., unlocked and loaded). Third, self-reported data might be subject to social desirability bias, especially given that surveys are conducted over the phone, which is likely to underestimate the prevalence of unsafe storage. Lastly, though we did not account for the impacts of CAP laws, existing research shows that firearm owners in states with stringent CAP laws were no more likely to lock firearm than those in states without those laws [42]. Additionally, we did not have information regarding firearm types, number/density of firearms in households, or reasons for owning firearms. These variables might also have affected safe firearm storage [13]. Firearm owners who owned both handguns and long guns– which are more commonly owned in rural areas [36]– tended to report storing firearms unlocked and unhidden more frequently than those who owned only handguns [37]. Further research is needed to examine potential risk factors, their interaction with contextual elements such as rural firearm culture and norms, and the effectiveness of intervention programs tailored to specific settings.

## Conclusions

Our findings reveal that about one in three firearm-owning households kept their firearms loaded in both rural and urban areas. Rural firearm owners who reported storing at least one loaded firearm were more likely to keep them unlocked than urban firearm owners. Improving firearm storage practices is an integral part of firearm injury prevention. Given the unique firearm culture in the rural US, interventions should target specific needs of rural firearm owners to more effectively mitigate the risk of unintentional injuries, suicides, and unauthorized access.

## Electronic supplementary material

Supplementary Material 1

## Data Availability

The datasets analysed in the current study are available in the CDC’s BRFSS repository, https://www.cdc.gov/brfss/annual_data/annual_data.htm.

## Abbreviation

BRFSS: Behavioral Risk Factor Surveillance System
