# Supplementary material for: Firearm safe storage practices among firearm owners in rural and urban households
Source: Inj Epidemiol. 2025 Jun 13;12:33. doi: 10.1186/s40621-025-00587-9 (PMC12164122; doi:10.1186/s40621-025-00587-9)
Supplement: Supplementary file 1 — Supplementary Material 1 [file 40621_2025_587_MOESM1_ESM.docx]

**Appendix A. Adjusted relative risk (ARR) with 95% confidence intervals examining the association between demographic characteristics and risk factors and storing firearms loaded compared to unloaded.**

|  | ARR | 95% CI |
| --- | --- | --- |
| **Rural counties (ref: urban counties)** | 1.00 | 0.93-1.06 |
| **Age group (ref: 18-24)** |  |  |
| 25-34 | 1.29 | 1.14-1.46 |
| 35-44 | 1.27 | 1.12-1.44 |
| 45-54 | 1.25 | 1.11-1.42 |
| 55-64 | 1.15 | 1.01-1.31 |
| 65+ | 1.11 | 0.97-1.28 |
| **Female (ref: male)** | 0.70 | 0.67-0.74 |
| **Marital status (ref: married)** |  |  |
| Divorced/separated | 1.21 | 1.14-1.28 |
| Widowed | 1.19 | 1.10-1.28 |
| Never married | 1.01 | 0.94-1.10 |
| Unmarried couple | 1.08 | 0.97-1.21 |
| **Education attainment (ref: less than high school)** |  |  |
| Graduated high school | 1.15 | 1.03-1.29 |
| Attended college or technical school | 1.15 | 1.02-1.29 |
| Graduated from college or technical school | 0.96 | 0.86-1.08 |
| **Race/ethnicity (ref: Non-Hispanic White)** |  |  |
| Non-Hispanic Black | 1.18 | 1.10-1.27 |
| Non-Hispanic other race | 1.03 | 0.92-1.15 |
| Hispanic | 0.90 | 0.80-1.01 |
| **Ever served on active duty in the US military (ref: No)** | 1.15 | 1.09-1.22 |
| **Employment status (ref: employed or self-employed)** |  |  |
| Out of work | 0.86 | 0.75-0.99 |
| Homemakers | 0.79 | 0.66-0.94 |
| Students | 0.72 | 0.57-0.90 |
| Retired | 0.98 | 0.91-1.06 |
| Unable to work | 1.11 | 1.00-1.22 |
| **Any children in household (ref: No)** | 0.80 | 0.75-0.86 |
| **Any binge drinking in past 30 days (ref: No or N/A)** | 1.20 | 1.13-1.27 |
| **State (ref: Alaska)** |  |  |
| Arizona | 1.10 | 0.88-1.38 |
| California | 0.73 | 0.61-0.87 |
| Idaho | 0.91 | 0.72-1.14 |
| Indiana | 1.07 | 0.86-1.34 |
| Louisiana | 1.20 | 0.96-1.51 |
| Michigan | 0.70 | 0.55-0.88 |
| Minnesota | 0.56 | 0.48-0.66 |
| Nevada | 1.15 | 0.96-1.38 |
| New Jersey | 0.47 | 0.35-0.64 |
| New Mexico | 1.11 | 0.97-1.28 |
| North Carolina | 1.44 | 1.28-1.61 |
| Ohio | 1.10 | 0.98-1.23 |
| Oklahoma | 1.38 | 1.24-1.55 |
| Oregon | 0.72 | 0.57-0.91 |
| South Carolina | 1.02 | 0.82-1.27 |
| Vermont | 0.46 | 0.36-0.59 |
| Virginia | 1.01 | 0.80-1.27 |
| West Virginia | 1.09 | 0.87-1.36 |
| **Interview year (ref: 2021)** |  |  |
| 2022 | 1.12 | 1.00-1.26 |
| 2023 | 1.19 | 0.98-1.44 |
| 2024 | 1.15 | 0.92-1.43 |

ARR = adjusted relative risk.

**Appendix B. Adjusted relative risk (ARR) with 95% confidence intervals examining the association between demographic characteristics and risk factors and storing firearms locked compared to unlocked among those who stored firearms loaded.**

|  | ARR | 95% CI |
| --- | --- | --- |
| **Rural counties (ref: urban counties)** | 1.11 | 1.03-1.19 |
| **Age group (ref: 18-24)** |  |  |
| 25-34 | 1.15 | 0.97-1.36 |
| 35-44 | 1.08 | 0.91-1.28 |
| 45-54 | 1.21 | 1.03-1.42 |
| 55-64 | 1.20 | 1.01-1.41 |
| 65+ | 1.28 | 1.07-1.52 |
| **Female (ref: male)** | 0.82 | 0.77-0.88 |
| **Marital status (ref: married)** |  |  |
| Divorced/separated | 1.11 | 1.04-1.18 |
| Widowed | 1.13 | 1.05-1.22 |
| Never married | 1.12 | 1.02-1.24 |
| Unmarried couple | 1.07 | 0.94-1.23 |
| **Education attainment (ref: less than high school)** |  |  |
| Graduated high school | 0.93 | 0.82-1.05 |
| Attended college or technical school | 0.93 | 0.82-1.05 |
| Graduated from college or technical school | 0.87 | 0.77-.99 |
| **Race/ethnicity (ref: Non-Hispanic White)** |  |  |
| Non-Hispanic Black | 1.00 | 0.91-1.10 |
| Non-Hispanic other race | 0.89 | 0.76-1.05 |
| Hispanic | 1.06 | 0.92-1.22 |
| **Ever served on active duty in the US military (ref: No)** | 0.98 | 0.92-1.06 |
| **Employment status (ref: employed or self-employed)** |  |  |
| Out of work | 1.04 | 0.90-1.21 |
| Homemakers | 0.99 | 0.75-1.32 |
| Students | 0.86 | 0.60-1.23 |
| Retired | 1.10 | 1.02-1.19 |
| Unable to work | 1.06 | 0.94-1.20 |
| **Any children in household (ref: No)** | 0.64 | 0.58-0.71 |
| **Any binge drinking in past 30 days (ref: No or N/A)** | 1.18 | 1.10-1.27 |
| **State (ref: Alaska)** |  |  |
| Arizona | 0.85 | 0.64-1.12 |
| California | 0.75 | 0.60-0.92 |
| Idaho | 0.92 | 0.70-1.21 |
| Indiana | 0.78 | 0.60-1.03 |
| Louisiana | 0.88 | 0.66-1.16 |
| Michigan | 0.82 | 0.62-1.08 |
| Minnesota | 0.82 | 0.69-0.99 |
| Nevada | 0.77 | 0.62-0.96 |
| New Jersey | 0.49 | 0.32-0.75 |
| New Mexico | 0.80 | 0.68-0.94 |
| North Carolina | 0.88 | 0.77-1.00 |
| Ohio | 0.79 | 0.69-0.90 |
| Oklahoma | 0.98 | 0.87-1.10 |
| Oregon | 0.72 | 0.53-0.96 |
| South Carolina | 0.89 | 0.68-1.17 |
| Vermont | 0.88 | 0.66-1.17 |
| Virginia | 0.77 | 0.58-1.02 |
| West Virginia | 0.84 | 0.64-1.10 |
| **Interview year (ref: 2021)** |  |  |
| 2022 | 1.09 | 0.95-1.25 |
| 2023 | 1.03 | 0.80-1.32 |
| 2024 | 1.00 | 0.76-1.33 |

ARR = adjusted relative risk.

**Appendix C. Unadjusted and adjusted relative risk with 95% confidence intervals examining the association between firearm storage practices and rurality using the metropolitan and non-metropolitan indicator.**

|  | Loaded vs. unloaded | | | | Unlocked vs. locked | | | |
| --- | --- | --- | --- | --- | --- | --- | --- | --- |
|  | RR | 95% CI | ARR | 95% CI | RR | 95% CI | ARR | 95% CI |
| Metropolitan (reference) | - | - | - | - | - | - | - | - |
| Non-metropolitan | 1.01 | 0.96-1.06 | 0.98 | 0.93-1.02 | 1.16*** | 1.10-1.22 | 1.11*** | 1.06-1.17 |
| *Unweighted N* | 58,659 | | 57,255 | | 19,814 | | 19,288 | |

*Note.* ****p* <.001. RR = relative risk, ARR = adjusted relative risk (adjusted for sex, age, race/ethnicity, marital status, education attainment, employment status, military service, children in home, binge drinking, state, and interview year). The sample is limited to those who stored their firearms loaded in the unlocked vs. locked models.
